# Supplementary material for: Chromothripsis during telomere crisis is independent of NHEJ, and consistent with a replicative origin
Source: Genome Res. 2019 May;29(5):737–49. doi: 10.1101/gr.240705.118 (PMC6499312; doi:10.1101/gr.240705.118)
Supplement: Supplemental Material [file supp_gr.240705.118_Supplemental_file_1.zip › contigs/annotated_contigs/DB115/contig.3.DB115_length_830_mean_cov_7.95180722892.docx]

**DB115_length_830_mean_cov_7.95180722892**

```
GGATGGGGAATGGAGAGTTAGTATTTAGTGGGTACAGAGTTTCAGTTTTACAAGATGAAAAGAGTTCTAGAGAAGGATAGTGGTGATGG
 >chr8:28517888-28518231 - E=1e-186 p=1e-02
TTGCACAACATTATGAATGTATTTAATACCACTGAACTGTACACTTAAAAGTGATTAAGATGATAAATTGTGTTATGTATATTTTAACA

CAATAAAAATTGGGAGTGTGTGTATGTGTATATATATATGTCTGTGTGTACACACACACATATATATATAATTGGGAGTGTGTGTGTAT

ATACAGTATGTGTATGTTTGTATGAGAGCTTAACGTACATACACTTGTGTACATGTCTACCTAACAACTTTTTTTT|TT|CTTCCCTCA
                                                                             >chr8:2846
AGAACTTATAATTTTGTAGGTGTGGGTGTATAAAAGTAATTATATAAGGTAAGATGAAATAAGTACTATAATGTGTGTACATTCAAAAG
4573-28464734 + E=5e-85 p=0e+00
AGTACTGGGGCTAGGCAC|GGTGGCTCACGCCTGTTATCCCAGCACTTTGGGAGGCCAAGGC|GGGTGGATAATTAGAGTCCAGGGGTT
                   >chr8:28520158-28520525 + E=5e-189
CGAGACCAGCCTGGGCAACATGGTGAAATCCTGTCTCTGTAAAAAATACAAAAATTAGCCAGGCGTGGTGGCACGTGCCTGTGGTCCCA

GCTACTGGGGAGGCTGAGGTGGGAGCTCCTTGAACCCAGGAGGTCCAGGCTGCAGTGAGCTGAGCTGCGATTGCACCATTGCCCAGTTG

TTTGAGGCTGCAGTGAACCACGATTGTGCCACTGCACTCTAGCCTGGAAGACAGGGTGAAACCCTGTCTCCAAATAATTAAAAAGTAGA

TAGAAATGGAAATAATTGTATTACCGTACTATC
```
